# Complete Genome Sequence of *Vibrio parahaemolyticus* Environmental Strain UCM-V493

**DOI:** 10.1128/genomeA.00159-14

**Published:** 2014-03-13

**Authors:** S. S. Kalburge, S. W. Polson, K. Boyd Crotty, L. Katz, M. Turnsek, C. L. Tarr, J. Martinez-Urtaza, E. F. Boyd

**Affiliations:** a Department of Biological Sciences, University of Delaware, Newark, Delaware, USA; b Centers for Disease Control and Prevention, Atlanta, Georgia, USA; c Department of Biology and Biochemistry, University of Bath, Bath, United Kingdom

## Abstract

*Vibrio parahaemolyticus* is the leading bacterial cause of seafood-related gastroenteritis in the world. Here, we report the complete genome sequence and annotation of an environmental strain of *V. parahaemolyticus*, UCM-V493, with the aim of understanding the differences between the clinical and environmental isolates of the bacteria. We also make some preliminary sequence comparisons with the clinical strain RIMD2210633.

## GENOME ANNOUNCEMENT

*Vibrio parahaemolyticus* is a moderately halophilic Gram-negative bacterium found in marine environments in association with plankton, fish, and shellfish (1–4). *V. parahaemolyticus* is the leading bacterial cause of seafood-related gastroenteritis, with the CDC estimating 45,000 cases of infection yearly in the United States alone (4–7). Pathogenic strains of *V. parahaemolyticus* are characterized by the presence of *tdh* and *trh* genes coding for the thermostable direct hemolysin (TDH) and TDH-related hemolysin (TRH), respectively (1, 8–11). The first complete genome sequence of a *V. parahaemolyticus* strain was announced for the pandemic clinical isolate RIMD2210633 (12). Genomic analysis of this strain revealed the presence of 7 genomic islands, VPaI-1 to VPaI-7, ranging from 10 kb to 81 kb in size (13). An extensively studied environmental isolate, *V. parahaemolyticus* BB22OP, was recently sequenced and found to lack 5 of the 7 genomic islands present in the clinical isolate (14). Here, we report the complete genome sequence of *V. parahaemolyticus* UCM-V493. This strain is an O2:K28 serovar isolated in 2002 from a sediment sample in Spain. It is a *tdh*-negative and *trh*-negative strain and lacks all 7 genomic islands present in the clinical isolate (13, 15, 16).

*V. parahaemolyticus* UCM-V493 was grown at 37°C overnight in Luria-Bertani (LB) broth (Fisher Scientific, Fair Lawn, NJ) (pH 7) supplemented with streptomycin (200 µg/ml), with the final NaCl (Fisher Scientific) concentration adjusted to 3%. Genomic DNA was isolated using the GNOME DNA isolation kit (MP Biomedicals, Solon, OH).

Single-molecule real-time (SMRT) sequencing was performed on the PacBio RS2 platform (Pacific Biosciences, Menlo Park, CA). *De novo* assembly was performed using the hierarchical genome assembly process (HGAP) on the PacBio SMRT portal (17), and a coverage of 75× was obtained. Additional sequencing was performed using the Illumina MiSeq platform and Nextera technology (Illumina, San Diego, CA). The Illumina reads were assembled using CG-Pipeline (18) and CLC Genomics Workbench software package version 6.0.4 (CLC bio, Aarhus, Denmark), and the resulting contigs were used to manually fill gaps with the MEGA5 software (19). The UCM-493 genome is composed of two circular chromosomes and a circular plasmid. Annotation was performed using MAKER2 (20) and the RAST server (21). Chromosome 1 is 3.446 Mb and contains 3,187 coding sequences (CDSs), chromosome 2 is 1.698 Mb and contains 1,557 CDSs, and the plasmid is 88.5 kb and contains 116 CDSs. The G+C content is 45.3% for chromosome 1, 45.6% for chromosome 2, and 40.8% for the plasmid.

UCM-V493 shares a high homology with the clinical strain RIMD2210633; >80% of the CDSs are similar in the two strains. The UCM-V493 genome also shows a high level of gene synteny compared to RIMD2210633. Two novel prophage elements were identified in the UCM-V493 genome using the PHAST search tool (22). The prophage element on chromosome 1 shows homology to filamentous phage VCY-φ found in environmental *Vibrio cholerae* strains, and the prophage element on chromosome 2 shows homology to filamentous phage VFJ of *V. cholerae*. A detailed comparative analysis of the UCM-V493 and the RIMD2210633 genomes will be published elsewhere.

### Nucleotide sequence accession numbers.

The complete, annotated genome sequence for *V. parahaemolyticus* strain UCM-V493 was deposited at NCBI under accession no. CP007004 (UCM-V493_Chromosome_1), CP007005 (UCM-V493_Chromosome_2), and CP007006 (UCM-V493_pVPUCMV_plasmid).
